# Biomarkers of cellular senescence in idiopathic pulmonary fibrosis

**DOI:** 10.1186/s12931-023-02403-8

**Published:** 2023-04-07

**Authors:** Zaira Aversa, Elizabeth J. Atkinson, Eva M. Carmona, Thomas A. White, Amanda A. Heeren, Sarah K. Jachim, Xu Zhang, Steven R. Cummings, Sergio E. Chiarella, Andrew H. Limper, Nathan K. LeBrasseur

**Affiliations:** 1 Robert and Arlene Kogod Center on Aging, Mayo Clinic, 200 First Street SW, Rochester, MN 55905 USA; 2 Department of Physical Medicine and Rehabilitation, Mayo Clinic, Rochester, MN USA; 3 Department of Quantitative Health Sciences, Mayo Clinic, Rochester, MN USA; 4 Division of Pulmonary and Critical Care Medicine, Department of Medicine, Mayo Clinic, Rochester, MN USA; 5 Mayo Clinic Graduate School of Biomedical Sciences, Rochester, MN USA; 6 Departments of Medicine, Epidemiology and Biostatistics, University of California San Francisco, San Francisco, CA USA; 7 Research Institute, California Pacific Medical Center, San Francisco, CA USA; 8 Division of Allergic Diseases, Mayo Clinic, Rochester, MN USA

**Keywords:** Aging, Biomarkers, Cellular senescence, Idiopathic pulmonary fibrosis (IPF)

## Abstract

**Background:**

Cellular senescence is a cell fate in response to diverse forms of age-related damage and stress that has been implicated in the pathogenesis of idiopathic pulmonary fibrosis (IPF). The associations between circulating levels of candidate senescence biomarkers and disease outcomes have not been specifically studied in IPF. In this study we assessed the circulating levels of candidate senescence biomarkers in individuals affected by IPF and controls and evaluated their ability to predict disease outcomes.

**Methods:**

We measured the plasma concentrations of 32 proteins associated with senescence in Lung Tissue Research Consortium participants and studied their relationship with the diagnosis of IPF, parameters of pulmonary and physical function, health-related quality of life, mortality, and lung tissue expression of *P16*, a prototypical marker of cellular senescence. A machine learning approach was used to evaluate the ability of combinatorial biomarker signatures to predict disease outcomes.

**Results:**

The circulating levels of several senescence biomarkers were significantly elevated in persons affected by IPF compared to controls. A subset of biomarkers accurately classified participants as having or not having the disease and was significantly correlated with measures of pulmonary function, health-related quality of life and, to an extent, physical function. An exploratory analysis revealed senescence biomarkers were also associated with mortality in IPF participants. Finally, the plasma concentrations of several biomarkers were associated with their expression levels in lung tissue as well as the expression of *P16*.

**Conclusions:**

Our results suggest that circulating levels of candidate senescence biomarkers are informative of disease status, pulmonary and physical function, and health-related quality of life. Additional studies are needed to validate the combinatorial biomarkers signatures that emerged using a machine learning approach.

**Supplementary Information:**

The online version contains supplementary material available at 10.1186/s12931-023-02403-8.

## Introduction

Idiopathic pulmonary fibrosis (IPF) is a progressive and fatal interstitial lung disease for which limited therapeutic options exist. As the name implies, the underlying mechanisms of IPF are incompletely understood, which poses significant challenges for diagnosis and management. The heterogenous nature of IPF further complicates early detection, prediction of disease progression, and patient selection for and evaluation of response to existing and emerging therapies [1–3]. Hence, there is considerable interest in identifying accessible, reliable, and informative biomarkers of IPF [4].

Advanced chronological age is a primary risk factor for IPF. Etiological hallmarks of the disease, including aberrant repair and remodeling of the lung interstitium, mirror accelerated aging [5]. Cellular senescence is a cell fate in response to diverse forms of age-related damage and stress. Senescent cells are characterized by permanent growth arrest, resistance to apoptosis, and acquisition of a robust senescence-associated secretory phenotype (SASP) comprised of cytokines, chemokines, matrix metalloproteinases, and growth factors [6, 7]. A growing body of evidence suggests that the progressive accumulation of senescent fibroblasts and alveolar epithelial cells contribute to the pathogenesis of IPF and represent a potentially targetable mechanism [8–10]. Moreover, since the SASP can exert detrimental effects both locally and systemically [11–13], circulating concentrations of its components can be exploited as accessible biomarkers of senescent cell burden in an organism [14, 15]. However, the associations between candidate SASP components, the absence/presence of disease, clinically important measures, and patient-centered outcomes, have not yet been carefully examined in the context of IPF.

In this study, we measured the circulating levels of candidate senescence biomarkers in Lung Tissue Research Consortium (LTRC) participants and studied their relationship with the diagnosis of IPF, parameters of pulmonary and physical function, health-related quality of life (QoL), mortality, and *P16*, a prototypical marker of cellular senescence in lung tissue biopsies. By leveraging a machine learning approach, we evaluated the ability of combinatorial biomarker signatures to predict disease outcomes and to also identify the individual biomarkers with the greatest importance.

## Methods

### Study participants

Plasma samples (collected in lithium heparin–coated tubes), clinical parameters, and microarray data were obtained from a subset of LTRC participants affected by IPF or unaffected by IPF (controls). IPF diagnosis was based on American Thoracic Society and European Respiratory Society criteria [16]. To the best of our knowledge, none of the patients in this cohort were receiving antifibrotic therapies. The Mayo Clinic Institutional Review Board approved all aspects of the current study. Informed consent was obtained from all participants in the LTRC, comprising Mayo Clinic, University of Colorado, University of Michigan, Temple University and University of Pittsburgh.

### Measurement of circulating senescence biomarkers

The plasma protein concentrations of ADAMTS13, eotaxin, Fas, GDF15, GROα, ICAM1, IL1α, IL6, IL7, IL8, IL10, IL15, MCP1, MDC, MIP1α, MIP1β, MMP1, MMP7, MMP9, MPO, OPN, PAI1, PARC, RAGE, RANTES, SOST, TARC, TNFR1, TNFR2, TNFα, and VEGFA were quantified using commercially available multiplex magnetic bead-based immunoassays (R&D Systems) on the Luminex xMAP multianalyte profiling platform and analyzed on MAGPIX System (Merck Millipore). Activin A concentration was determined by a Quantikine ELISA Kit (R&D Systems). Protein names, abbreviations, and aliases are provided in Additional file 1: Table S1. All assays were performed according to the manufacturer’s protocols. In instances where a biomarker was below the limit of detection in a participant sample, a value of half of the lowest measured value for that analyte was assigned.

### Clinical and molecular outcome measures

We used forced vital capacity (FVC), forced expiratory volume in 1 s (FEV1), and diffusing capacity of the lung for carbon monoxide (DLCO) as measures of pulmonary function; St. George Respiratory Questionnaire (SGRQ) as a measure of health-related QoL; the 12-Item Short Form Health Survey (SF-12) and the 6-minute walk test (6MWT) as measures of physical function. Deaths among IPF participants were identified from follow-up with the patient’s family or physician or by search of the national death registry. Microarray data for gene expression in lung tissue biopsies were based on the probes indicated in additional file 1: Table S2.

### Statistical methods

Continuous and categorical participant characteristics among the control and IPF groups were compared using the Kruskal-Wallis rank sum test and the χ2-test, respectively. Kaplan-Meier curve was used to summarize overall survival in the IPF group. Spearman’s correlation coefficients were used to summarize biomarker and functional data relationships. Univariate Cox regression models were used to assess the relationship between biomarkers and overall survival. Least absolute shrinkage and selection operator (LASSO) regression analysis was used to avoid over-fitting and select a minimum set of biomarkers for predicting the absence/presence of disease, clinically important measures, patient-centered outcomes, and *P16* expression in lung tissue biopsies. Cross-validation was used to identify the penalty that minimized the model error. Because the number of subjects is relatively small, the data could not be split into training and testing cohorts. Instead, cross-validation approaches were used to estimate overall model predictive performance (area under the receiver operating characteristic curve (AUC) for binary outcomes and R^2^ for continuous outcomes), which helps adjust for the optimism obtained when models are summarized using the same data as was used to fit the model. To ease the interpretation of biomarker coefficients, biomarkers were standardized (x-mean)/SD prior to inclusion in the models. The knn nearest neighbor approach was used to impute the occasional missing biomarker value. The R (4.0.3) software package and GraphPad Prism 9 were used for statistical analysis and generation of graphs. P < 0.05 was considered statistically significant.

## Results

### Participant demographic and clinical characteristics

We studied the clinical data and biospecimens of 180 LTRC participants, with 95 affected by IPF and 85 defined as controls (Table 1). The two groups were of similar age, body mass index (BMI), and smoking history, but a lower proportion of participants with IPF were women. Regarding health history (Additional file 1: Table S3), gastroesophageal reflux disease was more common in participants with IPF, while lung and other cancers were more common in the control participants. Other comorbidities were not different between the two groups.

Participants with IPF showed a significant reduction in FVC, DLCO, and FEV1 compared to controls (Table 1). Correspondingly, SGRQ scores were significantly higher in participants with IPF, suggestive of impaired health-related QoL. Modest differences were observed between groups for self-reported function using the SF-12 and performance-based physical function using the 6MWT. These clinical findings parallel those reported for the entire LTRC cohort [17].

**Table 1:** Demographic and Clinical Characteristics of IPF and Control Participants

	Control (n = 85)	IPF (n = 95)	p-value
**Age, years** mean ± SD	63.40 ± 10.48	64.81 ± 8.72	0.411^*^
**Sex, females** n (%)	**45 (52.9%)**	**33 (34.7%)**	**0.014** ^†^
**BMI, Kg/m** ^**2**^ Mean ± SD	29.51 ± 6.40	30.38 ± 5.90	0.202^*^
**Ever Smoked (> 100)** n (%)	5 (6.5%)	11 (11.8%)	0.236^†^
**Pack Years** Mean ± SD	36.75 ± 35.31	27.26 ± 21.98	0.289^*^
**FVC, % predicted** Mean ± SD	**94.51 ± 12.97**	**68.42 ± 14.59**	**< 0.001** ^*^
**DLCO, % predicted** Mean ± SD	**83.66 ± 16.62**	**52.67 ± 16.56**	**< 0.001** ^*^
**FEV1, % predicted** Mean ± SD	**95.20 ± 12.66**	**75.59 ± 16.05**	**< 0.001** ^*^
**SGRQ** Mean ± SD	**12.48 ± 15.52**	**40.03 ± 19.67**	**< 0.001** ^*^
**SF-12** Mean ± SD	**48.09 ± 11.28**	**38.15 ± 10.47**	**< 0.001** ^*^
**6MWT, meters** Mean ± SD	**418.18 ± 118.42**	**387.16 ± 89.15**	**0.004** ^*^

Abbreviations: BMI = Body Mass Index; FEV1 = Forced Expiratory Volume in 1 s; FVC = Forced Vital Capacity; DLCO = Diffusing Capacity of the Lung for Carbon Monoxide; LTRC = Lung Tissue Research Consortium; 6MWT = Six Minute Walk Test; SF-12 = 12-item Short Form Health Survey; SGRQ = St. George Respiratory Questionnaire.

^*^ p-value represents the comparison between IPF and control participants using the Kruskal-Wallis rank sum test.

^†^ p-value represents the comparison between IPF and control participants using the Pearson’s chi-squared test.

### Biomarkers of cellular senescence in participants with IPF and controls

We assessed the plasma concentrations of 32 proteins associated with senescent cells and the SASP [14]. Strikingly, participants with IPF had significantly higher levels of activin A, eotaxin, GDF15, ICAM1, IL7, IL10, MCP1, MDC, MMP1, MMP7, MPO, PARC, RANTES, and TARC than controls (Table 2). Conversely, the circulating concentrations of ADAMTS13 and RAGE were significantly lower in participants with IPF compared to controls.

**Table 2:** Circulating Biomarkers Measured in the Plasma of IPF and Control Participants

Protein	Control n = 85	IPF n = 95	p-value
Activin A	**355.3 ± 193.9**	**418.4 ± 183.9**	**0.009**
ADAMTS13	**932465.0 ± 431528.9**	**796302.1 ± 368128.0**	**0.022**
Eotaxin	**1148.9 ± 469.5**	**1440.8 ± 667.9**	**0.002**
Fas	8688.8 ± 6202.1	7706.4 ± 2753.8	0.389
GDF15	**1463.2 ± 1542.3**	**2050.8 ± 1343.6**	**< 0.001**
GROα	246.1 ± 311.3	198.0 ± 99.5	0.331
ICAM1	**238606.1 ± 183090.8**	**259886.9 ± 161879.8**	**0.007**
IL1α	6.7 ± 6.7	9.5 ± 15.0	0.112
IL6	10.3 ± 16.8	9.7 ± 7.7	0.219
IL7	**3.1 ± 1.9**	**3.7 ± 1.9**	**0.022**
IL8	6.8 ± 4.3	9.0 ± 14.8	0.134
IL10	**22.1 ± 20.6**	**35.0 ± 42.5**	**0.015**
IL15	1.5 ± 1.3	1.5 ± 1.2	0.616
MCP1	**279.6 ± 88.6**	**363.2 ± 123.8**	**< 0.001**
MDC	**454.8 ± 149.7**	**611.8 ± 215.3**	**< 0.001**
MIP1α	17.0 ± 16.6	22.1 ± 37.8	0.345
MIP1β	370.0 ± 1724.5	162.4 ± 59.5	0.629
MMP1	**764.3 ± 813.7**	**772.9 ± 433.2**	**0.017**
MMP7	**2875.3 ± 2516.3**	**3828.2 ± 1591.5**	**< 0.001**
MMP9	54523.0 ± 70865.2	49,906 ± 56381.2	0.582
MPO	**82905.8 ± 111974.0**	**95750.7 ± 86099.0**	**0.031**
OPN	72451.1 ± 75720.1	52389.8 ± 55664.1	0.086
PAI1	20178.6 ± 11816.0	22684.1 ± 13130.5	0.188
PARC	**53061.1 ± 32883.8**	**72900.6 ± 41964.7**	**< 0.001**
RAGE	**1732.7 ± 829.4**	**1153.8 ± 554.2**	**< 0.001**
RANTES	**23107.9 ± 15665.1**	**32923.4 ± 30574.4**	**0.004**
SOST	442.8 ± 223.6	402.9 ± 207.6	0.183
TARC	**433.1 ± 126.7**	**726.8 ± 720.0**	**< 0.001**
TNFα	9.2 ± 7.2	8.3 ± 5.3	0.466
TNFR1	1518.2 ± 658.4	1370.0 ± 515.7	0.232
TNFR2	3608.3 ± 1910.0	3312.8 ± 1670.1	0.275
VEGFA	42.3 ± 18.8	39.1 ± 16.2	0.380

Data expressed as mean ± SD.

p-value represents the comparison between IPF and control participants using the Kruskal-Wallis rank sum test.

**Fig. 1:**
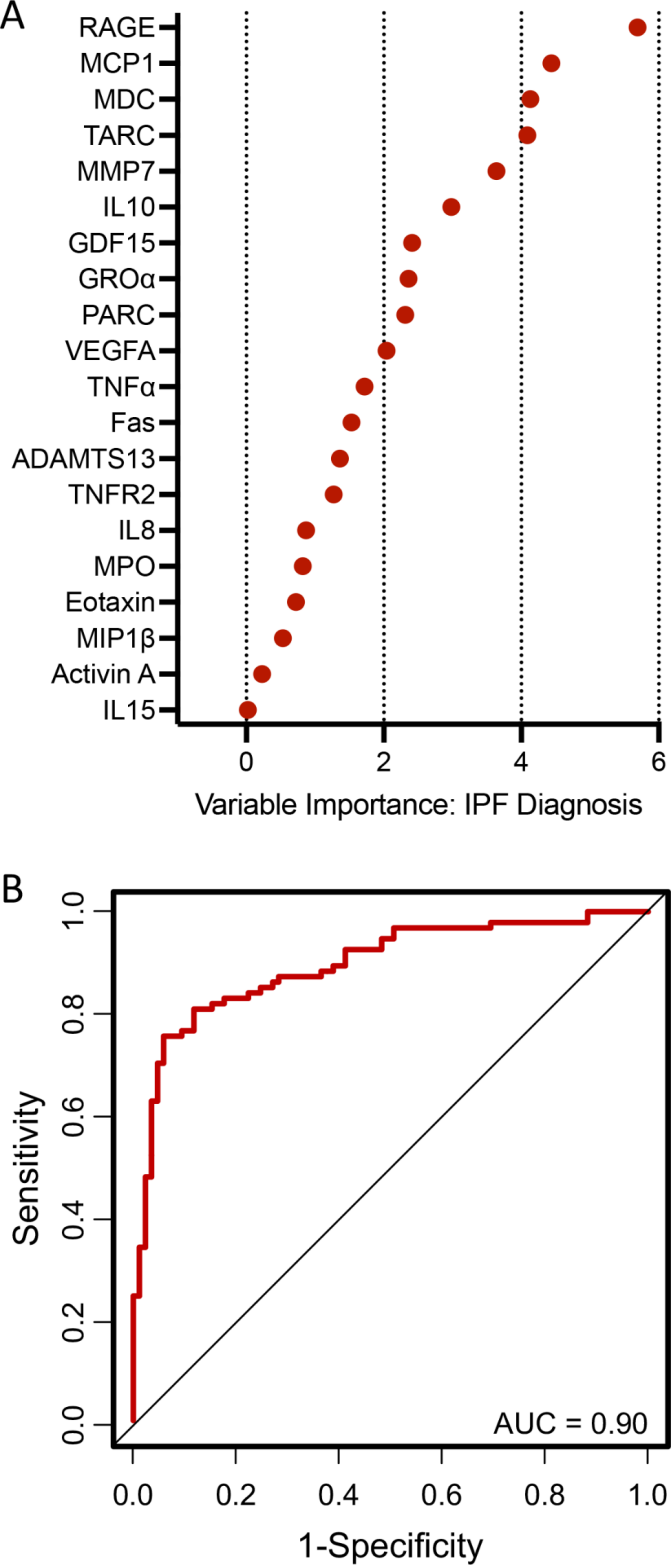
Biomarkers selected by the LASSO regression analysis to predict idiopathic pulmonary fibrosis (IPF) diagnosis (A) and area under the receiver operating characteristic curve (AUC) for this model (B)

To evaluate the ability of the biomarker panel to predict IPF cases, we next leveraged LASSO regression analysis, a machine learning approach, to also select the most important predictors. As summarized in Fig. 1A and Additional file 1 Table S4, the most important biomarkers in discerning participants with IPF from controls included RAGE, MCP1, MDC, TARC, MMP7, IL10, GDF15, GROα, PARC, and VEGFA. The AUC for the model to predict the absence or presence of disease was 0.90 (Fig. 1B), suggesting good discriminatory power.

### Associations of senescence biomarkers with clinical and patient-centered outcomes

**Fig. 2:**
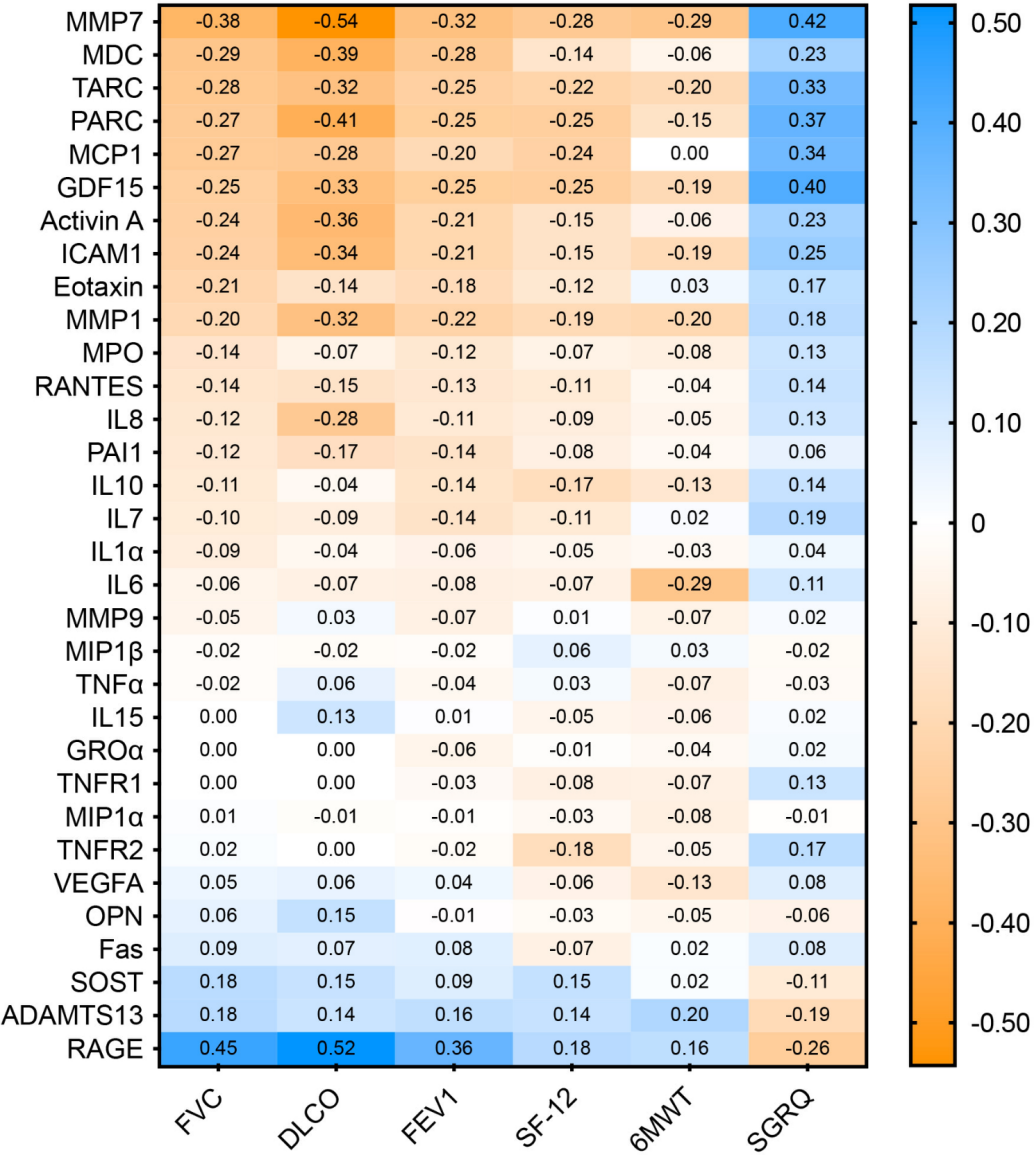
Heatmap representing the adjusted (for age, sex, and BMI) Spearman’s rank correlations between biomarkers and forced vital capacity (FVC, % predicted), diffusing capacity of the lung for carbon monoxide (DLCO, % predicted), forced expiratory volume in 1 s (FEV1, % predicted), St. George Respiratory Questionnaire (SGRQ), 12-Item Short Form Health Survey (SF-12), and 6-minute walk test (6MWT).

**Fig. 3:**
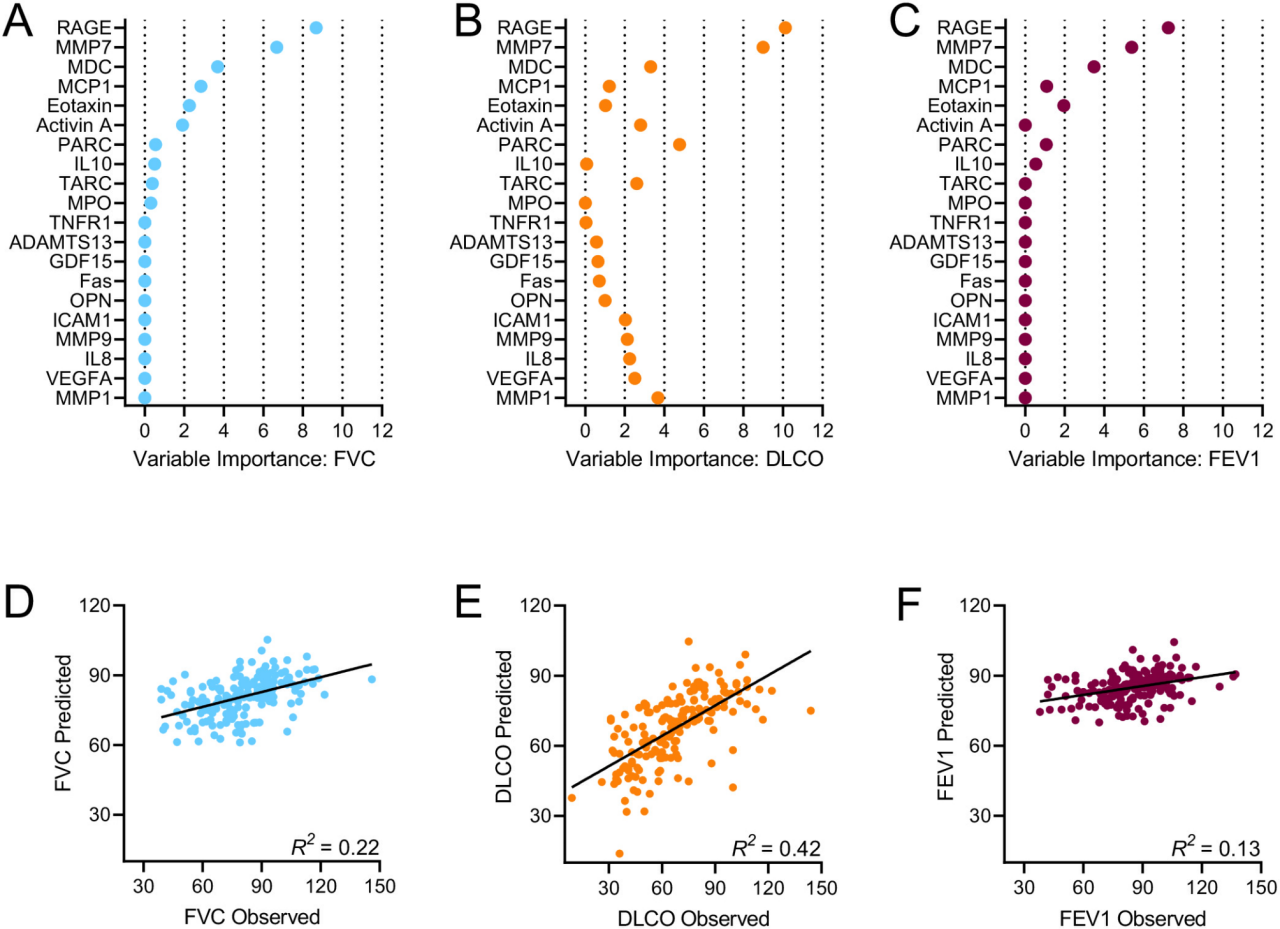
Biomarkers selected by the LASSO regression analysis to predict forced vital capacity (FVC, % predicted) (A), diffusing capacity of the lung for carbon monoxide (DLCO, % predicted) (B), and forced expiratory volume in 1 s (FEV1, % predicted) (C); Cross validation plots for the models predicting FVC (D), DLCO (E), and FEV1 (F)

We next evaluated the extent to which biomarkers were related to pulmonary function. Unadjusted Spearman’s rank correlations evidenced that numerous proteins were significantly associated with FVC, DLCO, and FEV1 (Additional file 1: Table S5-S7). The strongest associations were observed between FVC and RAGE (r = 0.46, p < 0.001), DLCO and MMP7 (r = -0.57, p < 0.001), and FEV1 and RAGE (r = 0.39, p < 0.001). After adjusting for age, sex, and BMI, the correlations between biomarkers and pulmonary function measures did not substantially change (Fig. 2, Additional file 1: Table S5-S7). The circulating concentrations of RAGE, MMP7, MDC, MCP1, eotaxin, and PARC emerged from LASSO regression models as important predictors of FVC, DLCO, and FEV1, and several additional senescence biomarkers (i.e., MMP1, MMP9, activin A, TARC, IL8, ICAM1, and VEGFA) were important determinants of DLCO (Fig. 3A-C, Additional file 1: Table S11). Based on cross validation, the model predicting DLCO had the highest correlation with the measured values (R^2^ = 0.42), followed by the models for FVC (R^2^ = 0.22), and FEV1 (R^2^ = 0.13) (Fig. 3D-F).

**Fig. 4:**
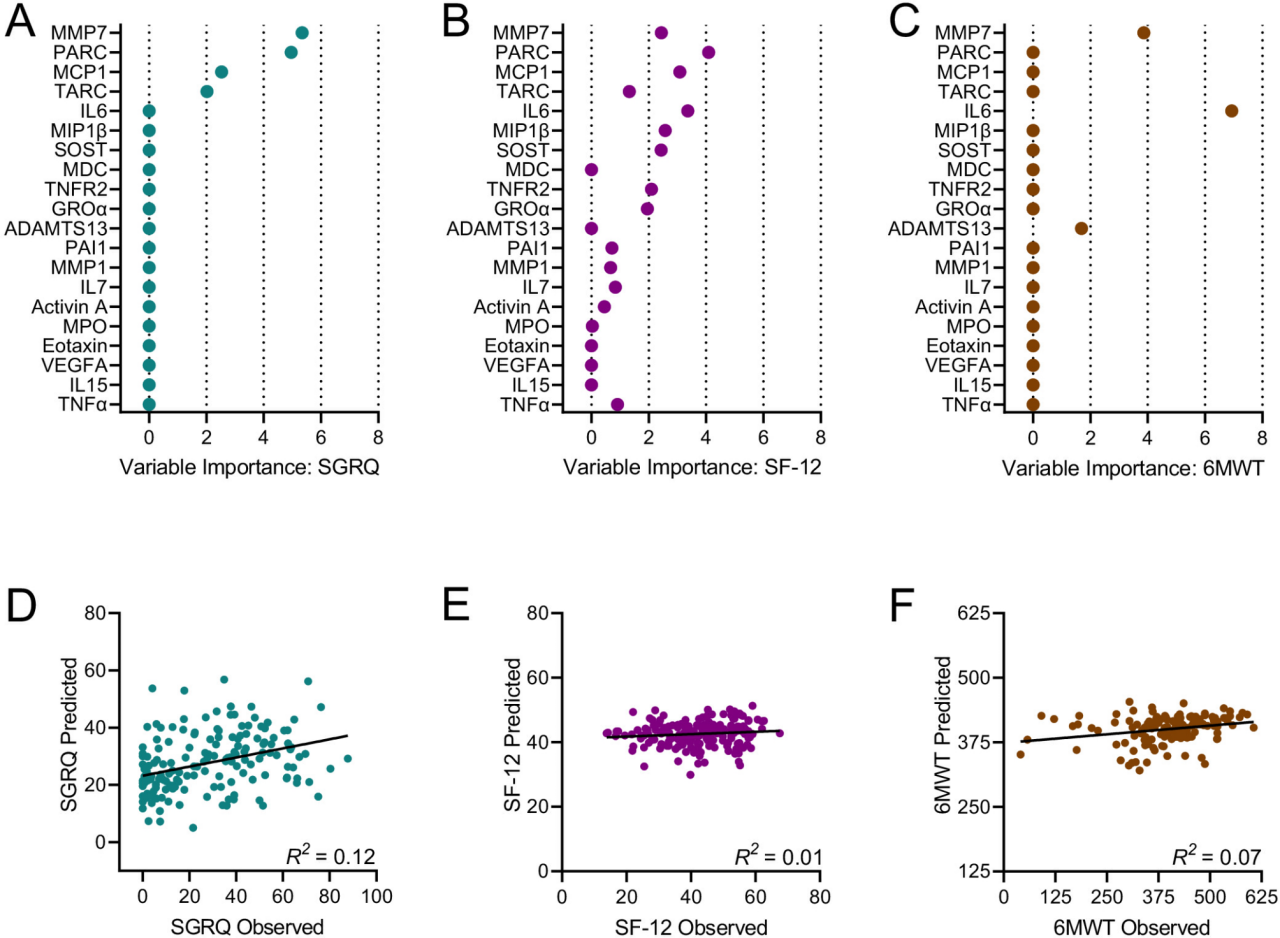
Biomarkers selected by the LASSO regression analysis to predict St. George Respiratory Questionnaire (SGRQ) (A), 12-Item Short Form Health Survey (SF-12) (B), and the 6-minute walk test (6MWT) (C); Cross validation plots for the models predicting SGRQ (D), SF-12 (E), and 6MWT (F)

Several biomarkers also demonstrated significant unadjusted and adjusted associations with health-related QoL (Additional file 1: Table S8). SGRQ scores were most strongly associated with plasma levels of PARC (r = 0.38, p < 0.001) and GDF15 (r = 0.38, p < 0.001). In LASSO regression models, MMP7, PARC, MCP1, and TARC levels were again identified as variables of greatest importance (Fig. 4A, Additional file 1: Table S12). The predictive model for SGRQ developed through cross-validation performed modestly (R^2^ = 0.12) (Fig. 4D).

Regarding self-reported function, a subset of the biomarkers showed significant unadjusted and adjusted associations with participant SF-12 scores (Additional file 1: Table S9). The association between plasma concentrations of MMP7 and SF-12 score was the strongest (r = -0.27, p < 0.001). The LASSO regression analysis distinguished PARC, IL6, MCP1, and MMP7 as important determinants of self-reported function, along with MIP1β, SOST, TNFR2, GROα, and TARC (Fig. 4B, Additional file 1: Table S12). However, the ability of biomarkers to predict SF-12 score was deemed poor in cross-validation models (R^2^ = 0.01) (Fig. 4E).

A subset of senescence biomarkers had significant unadjusted and adjusted associations with the 6MWT, a performance-based measure of physical function (Additional file 1: Table S10). IL6 was most strongly associated with distance walked (r = -0.30, p < 0.001). Only IL6, MMP7, and ADAMTS13 emerged as important predictors of 6MWT using LASSO regression (Fig. 4C, Additional file 1: Table S12), and the model for predicting walk distance performed poorly (cross-validated R^2^ = 0.07) (Fig. 4F).

**Fig. 5:**
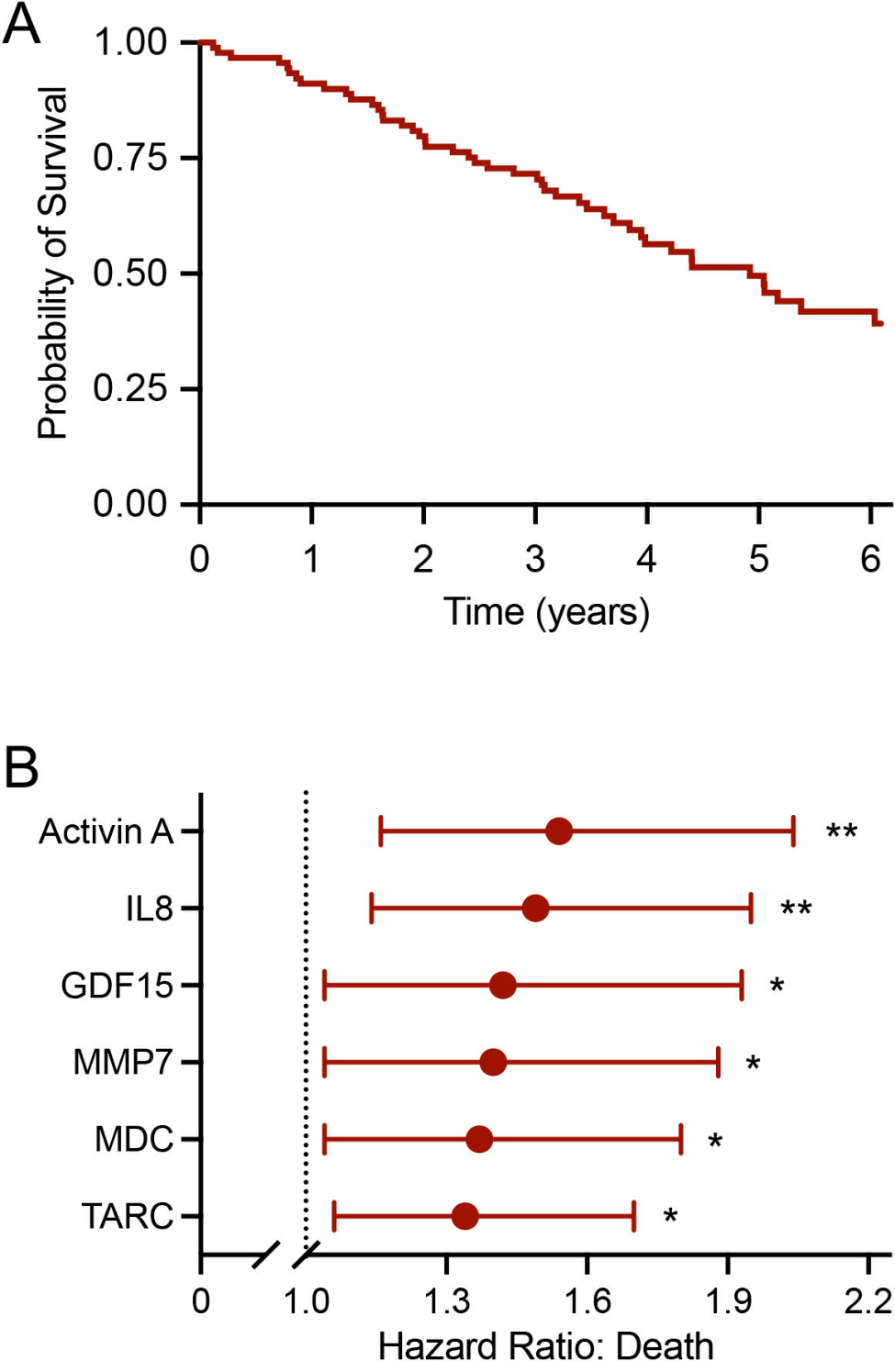
Survival curve for participants affected by idiopathic pulmonary fibrosis (IPF, n = 93) (A). Biomarkers associated with risk of death in participants with IPF identified by univariate Cox models adjusted for age, BMI, and sex (B). *p < 0.05 and **p < 0.01

Finally, as an exploratory analysis of study participants with IPF, we examined associations of senescence biomarkers and mortality. Over a 6.1-year follow-up period, 45 of 93 participants with IPF and follow-up data available for analysis died (Fig. 5A). Age, BMI, and sex did not exhibit associations with risk of death (all p-values for hazard ratios (HRs) > 0.693). Univariate Cox models adjusted for age, BMI, and sex demonstrated that higher concentrations of six senescence biomarkers (activin A, IL8, GDF15, MMP7, MDC, and TARC) were significantly associated with higher risk of death, with HRs ranging from 1.34 to 1.54 (all p < 0.026) (Fig. 5B, Additional file 1: Table S13).

### Association of plasma biomarkers of senescence with gene expression levels in the lung

**Fig. 6:**
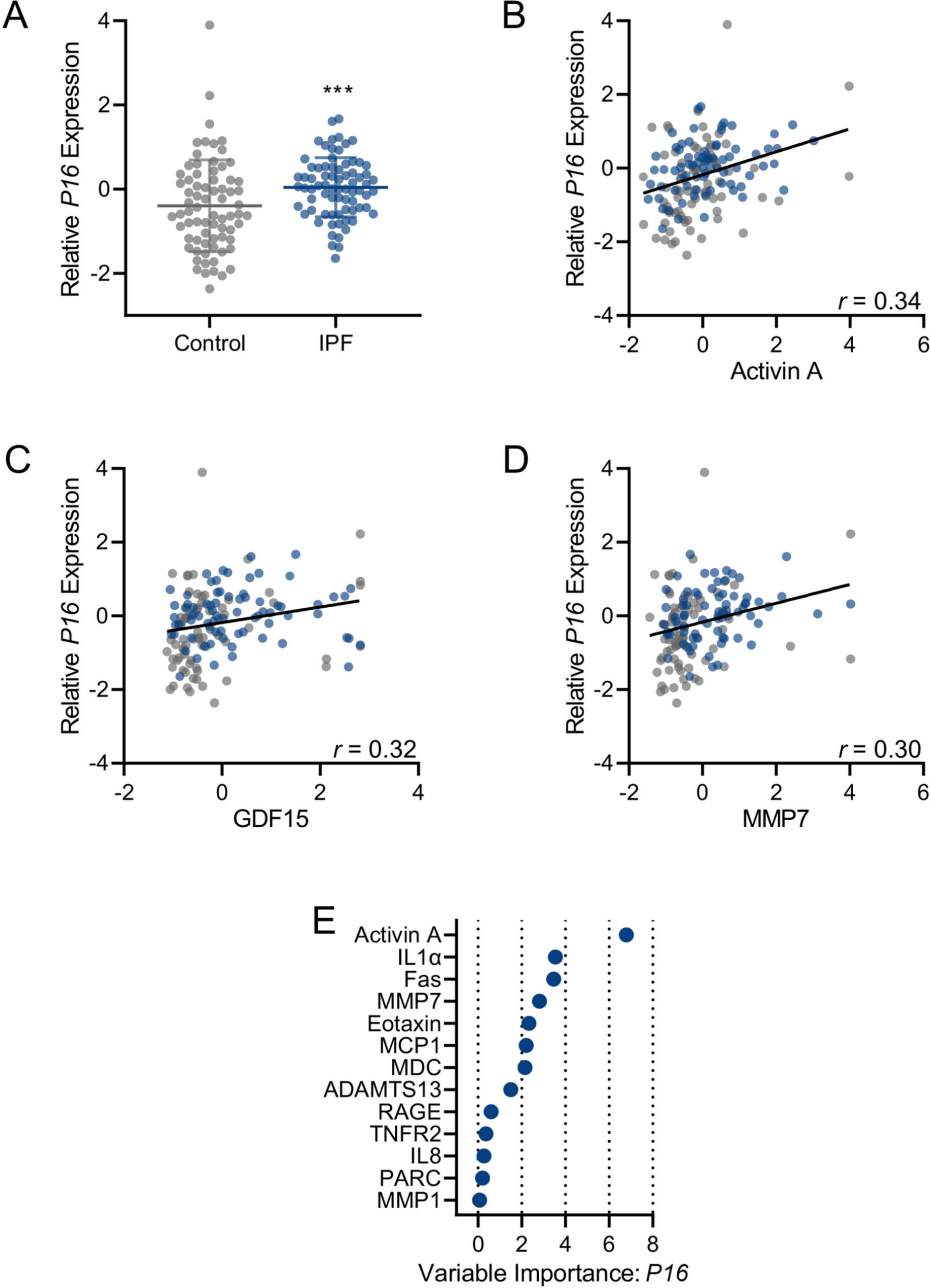
Lung *P16* gene expression levels in participants affected by idiopathic pulmonary fibrosis (IPF, n = 74) and controls (n = 73) captured through microarray analysis and expressed as means ± SD (A); Unadjusted Spearman’s rank correlations plots between lung *P16* gene expression and circulating levels of activin A (B), GDF15 (C), and MMP7 (D); Biomarkers selected by the LASSO regression analysis to predict lung *P16* gene expression (E).***p < 0.001

To assess whether circulating biomarker levels were associated with senescent cell burden in the lung tissue, we used expression levels of *P16* (*CDKN2A*) and *P21 (CDKN1A)*, prototypical markers of cellular senescence, previously captured through microarray analysis [8]. *P16* expression was significantly higher in lung biospecimens from participants with IPF compared to controls (Fig. 6A). The expression levels of *P21* were not different between groups (p = 0.513). Several circulating biomarkers of senescence were positively associated with *P16* expression before and after adjustment for age, BMI, and sex (Additional file 1: Table S14). The strongest associations observed were with activin A (r = 0.34, p < 0.001), GDF15 (r = 0.32, p < 0.001), and MMP7 (r = 0.30, p < 0.001) (Fig. 6B-D). Using LASSO regression, we identified activin A, IL1α, Fas, MMP7, eotaxin, MCP1, and MDC, as important predictors of lung *P16* expression levels (Fig. 6E, Additional file 1: Table S15). However, the cross-validated model R^2^ for predicting *P16* expression with this model was low (cross-validated R^2^ = 0.08).

**Fig. 7:**
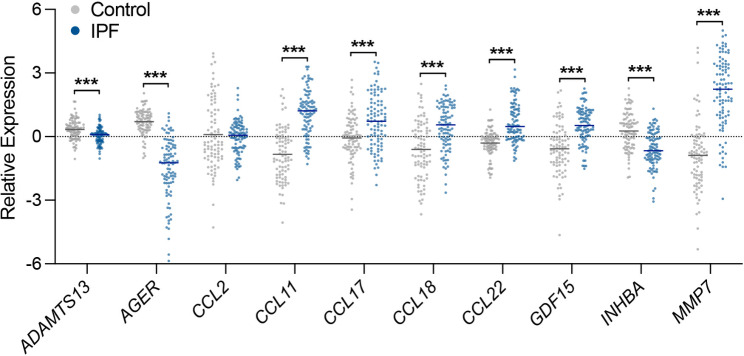
Lung gene expression levels (individual data points and means) of a subset of biomarkers in participants affected by idiopathic pulmonary fibrosis (IPF, n = 95) and controls (n = 85) captured through microarray analysis. ***p < 0.001

Consistent with higher levels of *P16* expression, levels of gene expression for our top biomarkers including *CCL11* (eotaxin), *CCL17* (TARC), *CCL18* (PARC), *CCL22* (MDC), *GDF15*, and *MMP7* were also significantly higher in lung biospecimens from IPF compared to control participants (all p < 0.001) (Fig. 7). In contrast, expression levels of *ADAMTS13*, *AGER* (RAGE), and *INHBA* (activin A) were significantly lower in the lung tissue biospecimens of IPF compared to control participants (all p < 0.001).

Finally, we examined whether the circulating abundance of our top biomarkers correlated with their gene expression levels in lung tissue biospecimens. We found that circulating protein concentrations of eotaxin, GDF15, MDC, MMP7, PARC, RAGE, and TARC were significantly and positively associated with their gene expression levels in the lung after adjusting for age, sex, and BMI (Table 3).

**Table 3:** Unadjusted and Adjusted (for age, sex, and BMI) Spearman’s Rank Correlations between a Subset of Circulating Biomarkers and Their Lung Gene Expression Levels Captured Through Microarray Analysis

Protein	Gene Symbol	Unadjusted r-value	Unadjusted p-value	Adjusted r-value	Adjusted p-value
Activin A	*INHBA*	-0.14	0.052	-0.13	0.074
ADAMTS13	*ADAMTS13*	0.05	0.489	0.05	0.536
Eotaxin	*CCL11*	**0.28**	**< 0.001**	**0.18**	**0.017**
GDF15	*GDF15*	**0.24**	**0.001**	**0.21**	**0.005**
MCP1	*CCL2*	0.11	0.144	0.10	0.172
MDC	*CCL22*	**0.29**	**< 0.001**	**0.28**	**< 0.001**
MMP7	*MMP7*	**0.44**	**< 0.001**	**0.42**	**< 0.001**
PARC	*CCL18*	**0.34**	**< 0.001**	**0.30**	**< 0.001**
RAGE	*AGER*	**0.38**	**< 0.001**	**0.39**	**< 0.001**
TARC	*CCL17*	**0.32**	**< 0.001**	**0.26**	**< 0.001**

## Discussion

In this study, we found that circulating biomarkers of cellular senescence, a cell fate implicated in the biology of aging and aging-related diseases, are significantly elevated in persons affected by IPF. Using a machine learning approach, we identified a subset of biomarkers, including RAGE, MCP1, MDC, TARC, MMP7, IL10, GDF15, GROα, PARC, and VEGFA, that accurately classified participants as having or not having the disease and contributed to a model that displayed impressive discriminatory power at cross-validation. Correspondingly, these senescence biomarkers were significantly correlated with measures of pulmonary function, health-related QoL and, to an extent, physical function. The plasma concentrations of several biomarkers (activin A, IL8, GDF15, MMP7, MDC, and TARC) were also significantly associated with risk of death in IPF participants after adjusting for age, BMI, and sex. Importantly, we observed that circulating concentrations of several biomarkers were associated with their corresponding gene expression levels in lung tissue as well as the expression of *P16*, a well-defined marker of senescent cells. Our data support the premise that cellular senescence is a hallmark of IPF and circulating components of the SASP may be of utility in both clinical practice and clinical research.

Transcriptional upregulation of core senescence markers, including the cyclin-dependent kinase inhibitor *P16* and several SASP components, has been reported in experimental murine models of lung fibrosis [8–10]. Results from these studies have also indicated that both fibroblasts and alveolar epithelial cells are prone to senesce in the fibrotic lung, that the robust and dynamic secretome of senescent fibroblasts exerts profibrotic effects *in vitro*, and both genetic and pharmacologic elimination of senescent cells improves parameters of pulmonary and physical function. Importantly, a significant increase in *P16* expression in the lung tissue biopsies of individuals affected by IPF obtained from the LTRC as well as from the European IPF registry has been previously reported and found to be negatively correlated with measures of pulmonary function [8, 10]. Collectively, these data support the premise that components of the SASP could serve as biomarkers of senescent cell burden in persons with IPF and be informative of disease status.

The candidate panel of senescence biomarkers we used in the current study was based on diverse components of the SASP commonly observed in cell-based models of senescence or in murine tissues with high senescent cell burden consequent to chronological aging or manipulation to induce an aging-related disease. While individual components of our biomarker panel are certainly not unique to senescent cells, we recently demonstrated that most of the cytokines, chemokines, matrix remodeling proteins, and growth factors measured herein are robustly secreted by a variety of human cell types driven to senesce in vitro, and their circulating concentrations significantly *increase* with chronological age in humans, consistent with an age-related increase in senescent cell burden [14]. An exception is RAGE, which was significantly *reduced* in participants with IPF and, in LASSO regression models, emerged as the most important biomarker in predicting case status, all three measures of pulmonary function (FVC, DLCO, and FEV1), and health-related QoL. RAGE is a member of the immunoglobulin superfamily of receptors highly expressed in the lung that exists in a membrane-bound form as well as in a soluble form, which acts as a decoy receptor [18]. Consistent with our results, a previous study found reduced plasma soluble RAGE in persons with IPF and other ILDs and a significant association with disease severity [19]. Interestingly, a nuclear isoform of RAGE has been implicated in DNA repair, and RAGE knockout mice develop fibrosis and exhibit markers of cellular senescence [20]. In the present study, we did not observe an association of RAGE with lung tissue expression of *P16*, although the combinatorial signature of biomarkers predicting *P16* lung expression included RAGE. The role of RAGE in the pathogenesis of IPF, however, is still not fully understood and conflicting results have been reported from murine models of lung fibrosis, underscoring the need of additional studies [18].

The higher expression of *P16* in human lung tissue with advanced IPF disease severity is paralleled by an increase in the expression of several matrix-remodeling proteins [8]. MMP7 is a metalloprotease that has been implicated in the pathogenesis of pulmonary fibrosis [21]. Here, the plasma concentration of MMP7 emerged as an important predictor of case status, pulmonary function, health-related QoL, and self-reported physical function; findings consistent with prior studies supporting MMP7 as a biomarker for IPF [22–27]. Our data further advance this concept by demonstrating a significant association between the circulating concentrations of MMP7 and lung *P16* expression, suggesting a plausible mechanism for the elevated abundance of this biomarker in the present as well as prior studies.

In addition to RAGE and MMP7, the plasma concentrations of activin A, eotaxin, MCP1, MDC, PARC, and TARC were also identified as predictors of key disease outcomes. Remarkably, like MMP7, levels of activin A, eotaxin, MCP1, MDC, and PARC were also significantly associated with lung *P16* expression and the LASSO regression designated activin A as the most important predictor. A recent study found increased circulating levels of activin A in persons with acute exacerbation of IPF compared to those with stable IPF and, similar to our study, evidenced a significant negative correlation with DLCO [28]. As our data would suggest, the plasma concentration of activin A is an important predictor of lung senescent cell burden, an interesting future direction would be to explore the contribution of senescent cells and the SASP to acute IPF exacerbation.

GDF15, a stress-induced cytokine, has emerged as a promising biomarker of chronological and biological aging and is a component of the SASP [14, 29, 30]. In the present study, we observed that plasma concentrations of GDF15 were significantly elevated in participants with IPF and associated with clinical and patient-centered outcomes as well as lung *P16* expression. With machine learning, GDF15 was most prominent in predicting the absence or presence of the disease and, to a lesser extent, DLCO and health-related QoL. In line with our findings, a previous study reported increased levels of GDF15 in the plasma of IPF patients from three independent cohorts and evidenced a significative negative correlation with DLCO [31]. In addition, higher GDF15 levels were recently associated with increased odds of interstitial lung abnormalities detected by chest computed tomography in two large independent cohorts [32]. Collectively, these data support GDF15 as an informative biomarker of health in persons with IPF, whether it reflects systemic or lung-specific aging.

Of the SASP components studied herein, IL6 is perhaps the most exhaustively studied in the context of aging and chronic disease [33]. Interestingly, circulating concentrations of IL6 did not differ between controls and participants with IPF. Even so, levels of IL6 were significantly associated with the 6MWT and this cytokine was selected as the most important predictor by the LASSO regression. This is consistent with prior studies of older adults that have further demonstrated that IL6 levels are informative of the risk of declines in gait speed [15, 34]. We note high levels of IL6 were previously detected in the secretome of senescent fibroblasts in vitro [8, 14] and a previous study found that circulating IL6 was significantly higher in persons affected by IPF compared to controls and that it was an independent predictor of DLCO decline [35]. Hence, in conjunction with the biomarkers discussed above, IL6 may be a useful biomarker of physical function and potentially pulmonary function in the context of IPF.

In clinical practice, biomarkers can potentially contribute to the diagnosis and management of IPF. As noted, they may reflect disease progression and even exacerbation and thus, guide clinical decision making. Reliable and accessible biomarkers of senescent cell burden may be also important for use in future clinical trials. There is now considerable interest in the development of senotherapeutic drugs, which can either eliminate (senolytics) or alter the behavior (senomorphics) of senescent cells. Pharmacological clearance of senescent cells has been demonstrated *in vitro* on both senescent fibroblasts and senescent alveolar epithelial cells by using the senolytic cocktail of dasatinib (a tyrosine kinase inhibitor) plus quercetin, (a flavonol) (DQ) [8, 10]. Consistently, administration of DQ to mice with bleomycin-induced lung fibrosis significantly reduced transcriptional levels of *P16* and several SASP factors in lung tissue and translated into improved measures of pulmonary and physical function in mice [8]. Encouragingly, no adverse events were reported in an open label pilot study in which the safety of a short duration intermittent treatment with DQ was evaluated in 14 participants with IPF [36]. Biomarkers offer utility to additional early phase trials as evidence of target engagement for optimization of dose and dosing regimens. Moreover, in ensuing randomized control trials to ultimately gauge efficacy and safety of senotherapeutics, biomarkers may be leveraged for the inclusion of participants who may be most responsive and serve as surrogate endpoints for longer-term outcomes of primary interest; i.e., FVC and DLCO. Enthusiasm for the senescence biomarkers presented here is consequent to their associations with disease status, pulmonary function, and, importantly, a contributing biological mechanism to IPF disease onset and/or progression.

Strengths of the present study include the standardized and methodically collected data elements and samples provided by the LTRC. The combination of lung tissue microarray data, plasma concentrations of senescence biomarkers, pulmonary and physical function measures, and health-related QoL are distinguishing features of our study that advance insights into IPF. We further note our observations may be strengthened by the health status of the control group, which was adversely influenced by smoking history and a higher frequency of lung and other cancers than participants with IPF. We also acknowledge study limitations. First, we used a pre-defined panel of candidate senescence biomarkers not biased toward lung disease. This may have limited our ability to capture other relevant biomarkers for IPF. Second, the cross-sectional nature of the study did not allow to further explore the temporal relationship between the circulating concentration of the biomarkers assessed and changes in clinical and patient-centered outcomes. Third, because of the unique elements in our data set, particularly access to lung tissue samples and data, we did not have a validation cohort to check the extent to which the associations and models’ predictions hold true in a different population. In light of this concern, we did perform cross-validation to qualify our primary findings. Fourth, we acknowledge that our candidate biomarkers, while informed by diverse experimental data, are neither unique nor universal to senescent cells. Through the analysis of previously captured microarray data, however, we observed increased lung gene expression of *P16* and of several of our top candidate senescence biomarkers in IPF participants compared to controls, which warrants further research.

In conclusion, our study demonstrates biomarkers of cellular senescence, a biological mechanism that may contribute to the etiology of IPF, are informative of disease status, pulmonary and physical function, health-related QoL, and mortality risk. Our data suggest senescence biomarkers may be of utility for clinical decision making, clinical research, and future trials of senotherapeutic interventions. Additional studies are warranted to validate and optimize the combinatorial signatures of biomarkers that emerged here using a machine learning approach.

## Electronic supplementary material

Below is the link to the electronic supplementary material.

**Additional file 1**: Table S1. List of the Proteins Measured in the Plasma of IPF and Control Participants. Table S2. Microarray probes. Table S3. Main Diagnoses Reported by IPF and Control Participants. Table S4. LASSO Regression Analysis to Predict Idiopathic Pulmonary Fibrosis (IPF) Diagnosis. Table S5. Unadjusted and Adjusted (for age, sex, and BMI) Spearman’s Rank Correlations between the Biomarkers and the Forced Vital Capacity (FVC). Table S6. Unadjusted and Adjusted (for age, sex, and BMI) Spearman’s Rank Correlations between the Biomarkers and Diffusing Capacity of the Lung for Carbon Monoxide (DLCO). Table S7. Unadjusted and Adjusted (for age, sex, and BMI) Spearman’s Rank Correlations between the Biomarkers and Forced Expiratory Volume in 1 s (FEV1). Table S8. Unadjusted and Adjusted (for age, sex, and BMI) Spearman’s Rank Correlations between the Biomarkers and St. George Respiratory Questionnaire (SGRQ). Table S9. Unadjusted and Adjusted (for age, sex, and BMI) Spearman’s Rank Correlations between the Biomarkers and 12-Item Short Form Health Survey (SF-12). Table S10. Unadjusted and Adjusted (for age, sex, and BMI) Spearman’s rank Correlations between the Biomarkers and 6-Minute Walk Test (6MWT). Table S11. LASSO Regression Analysis to Predict Forced Vital Capacity (FVC), Diffusing Capacity of the Lung for Carbon Monoxide (DLCO), and Forced Expiratory Volume in 1 s (FEV1). Table S12. LASSO Regression Analysis to Predict St. George Respiratory Questionnaire (SGRQ), 12-Item Short Form Health Survey (SF-12), and the 6-minute walk test (6MWT). Table S13: Associations between Circulating Biomarkers and Risk of Death after adjustment for Age, BMI, and Sex in IPF Participants. Table S14 Unadjusted and Adjusted (for age, sex, and BMI) Spearman’s Rank Correlations between the Circulating Biomarkers and *P16* Lung Gene Expression. Table S15. LASSO Regression Analysis to Predict Lung *P16* Gene Expression.

## Data Availability

The datasets used and/or analysed during the current study are available from the corresponding author on reasonable request.

## Abbreviations

AUC: Area under the receiver operating characteristic curve
BMI: Body mass index
DLCO: Diffusing capacity of the lung for carbon monoxide
FEV1: Forced expiratory volume in 1 s
FVC: Forced vital capacity
IPF: Idiopathic pulmonary fibrosis
LASSO: Least absolute shrinkage and selection operator
LTRC: Lung Tissue Research Consortium (LTRC)
6MWT: 6-minute walk test
QoL: Quality of life
SASP: Senescent associated secretory phenotype
SF-12: 12-Item Short Form Health Survey
SGRQ: St. George Respiratory Questionnaire
