# Supplementary material for: Biomarkers of cellular senescence in idiopathic pulmonary fibrosis
Source: Respir Res. 2023 Apr 7;24:101. doi: 10.1186/s12931-023-02403-8 (PMC10080755; doi:10.1186/s12931-023-02403-8)
Supplement: Supplementary file 1 — Additional file 1: Table S1. List of the Proteins Measured in the Plasma of IPF and Control Participants. Table S2. Microarray probes. Table S3. Main Diagnoses Reported by IPF and Control Participants. Table S4. LASSO Regression Analysis to Predict Idiopathic Pulmonary Fibrosis (IPF) Diagnosis. Table S5. Unadjusted and Adjusted (for age, sex, and BMI) Spearman’s Rank Correlations between the Biomarkers and the Forced Vital Capacity (FVC). Table S6. Unadjusted and Adjusted (for age, sex, and BMI) Spearman’s Rank Correlations between the Biomarkers and Diffusing Capacity of the Lung for Carbon Monoxide (DLCO). Table S7. Unadjusted and Adjusted (for age, sex, and BMI) Spearman’s Rank Correlations between the Biomarkers and Forced Expiratory Volume in 1 s (FEV1). Table S8. Unadjusted and Adjusted (for age, sex, and BMI) Spearman’s Rank Correlations between the Biomarkers and St. George Respiratory Questionnaire (SGRQ). Table S9. Unadjusted and Adjusted (for age, sex, and BMI) Spearman’s Rank Correlations between the Biomarkers and 12-Item Short Form Health Survey (SF-12). Table S10. Unadjusted and Adjusted (for age, sex, and BMI) Spearman’s rank Correlations between the Biomarkers and 6-Minute Walk Test (6MWT). Table S11. LASSO Regression Analysis to Predict Forced Vital Capacity (FVC), Diffusing Capacity of the Lung for Carbon Monoxide (DLCO), and Forced Expiratory Volume in 1 s (FEV1). Table S12. LASSO Regression Analysis to Predict St. George Respiratory Questionnaire (SGRQ), 12-Item Short Form Health Survey (SF-12), and the 6-minute walk test (6MWT). Table S13: Associations between Circulating Biomarkers and Risk of Death after adjustment for Age, BMI, and Sex in IPF Participants. Table S14 Unadjusted and Adjusted (for age, sex, and BMI) Spearman’s Rank Correlations between the Circulating Biomarkers and P16 Lung Gene Expression. Table S15. LASSO Regression Analysis to Predict Lung P16 Gene Expression. [file 12931_2023_2403_MOESM1_ESM.docx]

**ADDITIONAL FILE 1**

**Biomarkers of Cellular Senescence in Idiopathic Pulmonary Fibrosis**

Zaira Aversa, Elizabeth J. Atkinson, Eva M. Carmona, Thomas A. White, Amanda A. Heeren, Sarah K. Jachim, Xu Zhang, Steven R. Cummings, Sergio E. Chiarella, Andrew H. Limper, Nathan K. LeBrasseur

**Table S1**: List of the Proteins Measured in the Plasma of IPF and Control Participants

| **Protein name** | **Protein full name** | **Alias** |
| --- | --- | --- |
| Activin A | Activin A | INHBA |
| ADAMTS13 | A disintegrin and metalloproteinase with thrombospondin motifs 13 | VWFCP |
| Eotaxin | Eotaxin | CCL11 |
| Fas | Tumor necrosis factor receptor superfamily member 6 | APT1, TNFRSF6 |
| GDF15 | Growth/differentiation factor 15 | MIC1, NAG1, NRG1 |
| GROα | Growth-regulated alpha protein | CXCL1 |
| ICAM1 | Intercellular adhesion molecule 1 | CD54 |
| IL1α | Interleukin 1 alpha |  |
| IL6 | Interleukin 6 | IFNB2 |
| IL7 | Interleukin 7 |  |
| IL8 | Interleukin 8 | CXCL8 |
| IL10 | Interleukin 10 |  |
| IL15 | Interleukin 15 |  |
| MCP1 | Monocyte chemotactic protein 1 | CCL2 |
| MDC | Macrophage-derived chemokine | CCL22, SCYA22 |
| MIP1α | Macrophage inflammatory protein 1 alpha | CCL3, SCYA3 |
| MIP1β | Macrophage inflammatory protein 1-beta | CCL4, SCYA4 |
| MMP1 | Matrix metalloproteinase 1 | Interstitial collagenase |
| MMP7 | Matrix metalloproteinase 7 | Matrilysin |
| MMP9 | Matrix metalloproteinase 9 | CLG4B |
| MPO | Myeloperoxidase |  |
| OPN | Osteopontin | SPP1 |
| PAI1 | Plasminogen activator inhibitor 1 | SERPINE1, PLANH1 |
| PARC | Pulmonary and activation-regulated chemokine | CCL18 |
| RAGE | Advanced glycosylation end product-specific receptor |  |
| RANTES | Regulated on Activation, Normal T Cell Expressed and Secreted | CCL5, SCYA5 |
| SOST | Sclerostin | DAND6 |
| TARC | Thymus and activation-regulated chemokine | CCL17, SCYA17 |
| TNFR1 | Tumor necrosis factor receptor 1 | TNFRSF1A, CD120a |
| TNFR2 | Tumor necrosis factor receptor 2 | TNFRSF1B |
| TNFα | Tumor necrosis factor alpha | TNFSF2 |
| VEGFA | Vascular endothelial growth factor A | VPF |

**Table S2:** Microarray Probes

| **Gene symbol** | **Probe number** |
| --- | --- |
| *ADAMTS13* | A_23_P393645 |
| *AGER* | A_23_P93360 |
| *CCL2* | A_23_P89431 |
| *CCL11* | A_23_P66635 |
| *CCL17* | A_23_P26325 |
| *CCL18* | A_23_P55270 |
| *CCL22* | A_24_P313418 |
| *CDKN1A* | A_24_P89457 |
| *CDKN2A* | A_33_P3411628 |
| *GDF15* | A_23_P16523 |
| *INHBA* | A_23_P122924 |
| *MMP7* | A_23_P52761 |

**Table S3**: Main Diagnoses Reported by IPF and Control Participants

|  | **Control**  (n=85) | **IPF**  (n=95) | **p-value** |
| --- | --- | --- | --- |
| Angina | 4 (5.4%) | 11 (11.8%) | 0.149 |
| Arrhythmia | 10 (14.1%) | 16 (17.8%) | 0.527 |
| Asthma | 5 (6.8%) | 11 (12.1%) | 0.261 |
| Cirrhosis, chronic liver disease | 1 (1.4%) | 4 (4.3%) | 0.267 |
| Diabetes | 6 (8.1%) | 12 (12.9%) | 0.321 |
| DVT | 6 (8.1%) | 9 (9.8%) | 0.708 |
| GERD | **20 (27.0%)** | **38 (42.2%)** | **0.043** |
| Heart failure | 4 (5.4%) | 9 (9.7%) | 0.306 |
| Hepatitis | 3 (4.1%) | 4 (4.3%) | 0.940 |
| HIV | 0 (0.0%) | 1 (1.1%) | 0.371 |
| Hyperlipidemia | 39 (53.4%) | 53 (58.2%) | 0.537 |
| Lung cancer | **23 (38.3%)** | **2 (2.2%)** | **< 0.001** |
| Lupus | 0 (0.0%) | 0 (0.0%) |  |
| Other cancer | **26 (35.6%)** | **7 (7.6%)** | **< 0.001** |
| Polymyositis | 0 (0.0%) | 0 (0.0%) |  |
| Pulmonary Hypertension | 0 (0.0%) | 2 (2.5%) | 0.177 |
| Renal Failure | 3 (4.1%) | 3 (3.3%) | 0.786 |
| Rheumatoid Arthritis | 4 (5.4%) | 7 (7.8%) | 0.546 |
| Scleroderma | 0 (0.0%) | 0 (0.0%) |  |

Abbreviations: DVT = Deep Vein Thrombosis; GERD = Gastroesophageal Reflux Disease; HIV = Human Immunodeficiency Virus.

p-value represents the comparison between control and IPF patients using the Pearson’s chi-squared test.

**Table S4**: LASSO Regression Analysis to Predict Idiopathic Pulmonary Fibrosis (IPF) Diagnosis

| **Protein** | **Coefficient** | **Importance** |
| --- | --- | --- |
| RAGE | -0.976 | 5.52 |
| MCP1 | 0.820 | 4.64 |
| MDC | 0.781 | 4.42 |
| TARC | 0.728 | 4.12 |
| MMP7 | 0.703 | 3.98 |
| IL10 | 0.602 | 3.40 |
| GDF15 | 0.464 | 2.62 |
| GROα | -0.453 | 2.56 |
| PARC | 0.365 | 2.07 |
| VEGFA | -0.322 | 1.82 |
| TNFα | -0.314 | 1.78 |
| Fas | -0.307 | 1.74 |
| TNFR2 | -0.266 | 1.50 |
| ADAMTS13 | -0.256 | 1.45 |
| IL8 | -0.224 | 1.27 |
| MPO | 0.194 | 1.10 |
| Eotaxin | 0.133 | 0.75 |
| MIP1β | -0.105 | 0.59 |
| IL15 | -0.067 | 0.38 |
| Activin A | 0.018 | 0.10 |
| TNFR1 | -0.016 | 0.09 |
| MMP9 | -0.002 | 0.01 |
| RANTES | 0.000 | 0.00 |
| OPN | 0.000 | 0.00 |
| IL7 | 0.000 | 0.00 |
| PAI1 | 0.000 | 0.00 |
| SOST | 0.000 | 0.00 |
| IL1α | 0.000 | 0.00 |
| ICAM1 | 0.000 | 0.00 |
| MIP1α | 0.000 | 0.00 |
| IL6 | 0.000 | 0.00 |
| MMP1 | 0.000 | 0.00 |

**Table S5**: Unadjusted and Adjusted (for Age, Sex, and BMI) Spearman’s Rank Correlations Between the Biomarkers and Forced Vital Capacity (FVC)

| **Protein** | **Unadjusted**  **r-value** | **Unadjusted**  **p-value** | **Adjusted**  **r-value** | **Adjusted**  **p-value** |
| --- | --- | --- | --- | --- |
| Activin A | **-0.22** | **0.003** | **-0.24** | **0.001** |
| ADAMTS13 | 0.11 | 0.127 | **0.18** | **0.017** |
| Eotaxin | **-0.24** | **0.001** | **-0.21** | **0.005** |
| Fas | 0.04 | 0.599 | 0.09 | 0.258 |
| GDF15 | **-0.24** | **0.001** | **-0.25** | **0.001** |
| GROα | 0.02 | 0.765 | 0.00 | 0.988 |
| ICAM1 | **-0.26** | **<0.001** | **-0.24** | **0.001** |
| IL1α | -0.09 | 0.255 | -0.09 | 0.211 |
| IL6 | -0.09 | 0.246 | -0.06 | 0.436 |
| IL7 | -0.14 | 0.067 | -0.10 | 0.173 |
| IL8 | -0.11 | 0.127 | -0.12 | 0.109 |
| IL10 | -0.10 | 0.170 | -0.11 | 0.149 |
| IL15 | 0.04 | 0.615 | 0.00 | 0.955 |
| MCP1 | **-0.28** | **<0.001** | **-0.27** | **<0.001** |
| MDC | **-0.30** | **<0.001** | **-0.29** | **<0.001** |
| MIP1α | -0.02 | 0.739 | 0.01 | 0.922 |
| MIP1β | -0.04 | 0.635 | -0.02 | 0.803 |
| MMP1 | **-0.19** | **0.013** | **-0.20** | **0.008** |
| MMP7 | **-0.38** | **<0.001** | **-0.38** | **<0.001** |
| MMP9 | -0.10 | 0.184 | -0.05 | 0.486 |
| MPO | -0.18 | 0.015 | -0.14 | 0.059 |
| OPN | 0.07 | 0.325 | 0.06 | 0.400 |
| PAI1 | **-0.16** | **0.037** | -0.12 | 0.118 |
| PARC | **-0.30** | **<0.001** | **-0.27** | **<0.001** |
| RAGE | **0.46** | **<0.001** | **0.45** | **<0.001** |
| RANTES | **-0.16** | **0.034** | -0.14 | 0.071 |
| SOST | 0.14 | 0.068 | **0.18** | **0.018** |
| TARC | **-0.30** | **<0.001** | **-0.28** | **<0.001** |
| TNFR1 | -0.04 | 0.552 | 0.00 | 0.999 |
| TNFR2 | -0.02 | 0.800 | 0.02 | 0.839 |
| TNFα | -0.02 | 0.807 | -0.02 | 0.807 |
| VEGFA | 0.05 | 0.508 | 0.05 | 0.536 |

**Table S6**: Unadjusted and Adjusted (for Age, Sex, and BMI) Spearman’s Rank Correlations Between the Biomarkers and Diffusing Capacity of the Lung for Carbon Monoxide (DLCO)

| **Protein** | **Unadjusted**  **r-value** | **Unadjusted**  **p-value** | **Adjusted**  **r-value** | **Adjusted**  **p-value** |
| --- | --- | --- | --- | --- |
| Activin A | **-0.38** | **<0.001** | **-0.36** | **<0.001** |
| ADAMTS13 | **0.17** | **0.031** | 0.14 | 0.078 |
| Eotaxin | **-0.22** | **0.005** | -0.14 | 0.065 |
| Fas | 0.02 | 0.795 | 0.07 | 0.346 |
| GDF15 | **-0.38** | **<0.001** | **-0.33** | **<0.001** |
| GROα | -0.04 | 0.616 | 0.00 | 0.977 |
| ICAM1 | **-0.36** | **<0.001** | **-0.34** | **<0.001** |
| IL1α | -0.05 | 0.518 | -0.04 | 0.604 |
| IL6 | -0.11 | 0.151 | -0.07 | 0.350 |
| IL7 | -0.10 | 0.186 | -0.09 | 0.236 |
| IL8 | **-0.30** | **<0.001** | **-0.28** | **<0.001** |
| IL10 | -0.08 | 0.307 | -0.04 | 0.633 |
| IL15 | 0.09 | 0.227 | 0.13 | 0.099 |
| MCP1 | **-0.30** | **<0.001** | **-0.28** | **<0.001** |
| MDC | **-0.37** | **<0.001** | **-0.39** | **<0.001** |
| MIP1α | -0.05 | 0.522 | -0.01 | 0.849 |
| MIP1β | -0.06 | 0.400 | -0.02 | 0.766 |
| MMP1 | **-0.33** | **<0.001** | **-0.32** | **<0.001** |
| MMP7 | **-0.57** | **<0.001** | **-0.54** | **<0.001** |
| MMP9 | 0.03 | 0.709 | 0.03 | 0.702 |
| MPO | -0.06 | 0.415 | -0.07 | 0.387 |
| OPN | 0.09 | 0.264 | 0.15 | 0.050 |
| PAI1 | **-0.16** | **0.033** | **-0.17** | **0.031** |
| PARC | **-0.43** | **<0.001** | **-0.41** | **<0.001** |
| RAGE | **0.50** | **<0.001** | **0.52** | **<0.001** |
| RANTES | -0.15 | 0.056 | -0.15 | 0.060 |
| SOST | 0.11 | 0.162 | 0.15 | 0.056 |
| TARC | **-0.37** | **<0.001** | **-0.32** | **<0.001** |
| TNFR1 | -0.03 | 0.667 | 0.00 | 0.953 |
| TNFR2 | -0.05 | 0.528 | 0.00 | 0.995 |
| TNFα | 0.04 | 0.642 | 0.06 | 0.426 |
| VEGFA | 0.01 | 0.926 | 0.06 | 0.443 |

**Table S7**: Unadjusted and Adjusted (for Age, Sex, and BMI) Spearman’s Rank Correlations Between the Biomarkers and Forced Expiratory Volume in 1 Second (FEV1)

| **Protein** | **Unadjusted**  **r-value** | **Unadjusted**  **p-value** | **Adjusted**  **r-value** | **Adjusted**  **p-value** |
| --- | --- | --- | --- | --- |
| Activin A | **-0.16** | **0.033** | **-0.21** | **0.005** |
| ADAMTS13 | 0.07 | 0.372 | **0.16** | **0.034** |
| Eotaxin | **-0.20** | **0.008** | **-0.18** | **0.014** |
| Fas | 0.05 | 0.508 | 0.08 | 0.308 |
| GDF15 | **-0.22** | **0.003** | **-0.25** | **0.001** |
| GROα | -0.01 | 0.885 | -0.06 | 0.433 |
| ICAM1 | **-0.22** | **0.003** | **-0.21** | **0.006** |
| IL1α | -0.06 | 0.455 | -0.06 | 0.393 |
| IL6 | -0.09 | 0.210 | -0.08 | 0.306 |
| IL7 | **-0.18** | **0.017** | -0.14 | 0.057 |
| IL8 | -0.10 | 0.190 | -0.11 | 0.132 |
| IL10 | -0.12 | 0.105 | -0.14 | 0.070 |
| IL15 | 0.06 | 0.430 | 0.01 | 0.938 |
| MCP1 | **-0.20** | **0.007** | **-0.20** | **0.009** |
| MDC | **-0.29** | **<0.001** | **-0.28** | **<0.001** |
| MIP1α | -0.04 | 0.625 | -0.01 | 0.901 |
| MIP1β | -0.04 | 0.637 | -0.02 | 0.789 |
| MMP1 | **-0.19** | **0.010** | **-0.22** | **0.003** |
| MMP7 | **-0.30** | **<0.001** | **-0.32** | **<0.001** |
| MMP9 | -0.13 | 0.078 | -0.07 | 0.333 |
| MPO | **-0.17** | **0.021** | -0.12 | 0.100 |
| OPN | 0.03 | 0.697 | -0.01 | 0.945 |
| PAI1 | **-0.18** | **0.014** | -0.14 | 0.057 |
| PARC | **-0.26** | **<0.001** | **-0.25** | **0.001** |
| RAGE | **0.39** | **<0.001** | **0.36** | **<0.001** |
| RANTES | **-0.15** | **0.042** | -0.13 | 0.097 |
| SOST | 0.05 | 0.509 | 0.09 | 0.242 |
| TARC | **-0.24** | **0.001** | **-0.25** | **0.001** |
| TNFR1 | -0.05 | 0.489 | -0.03 | 0.692 |
| TNFR2 | -0.03 | 0.663 | -0.02 | 0.771 |
| TNFα | -0.03 | 0.708 | -0.04 | 0.607 |
| VEGFA | 0.06 | 0.429 | 0.04 | 0.629 |

**Table S8**: Unadjusted and Adjusted (for Age, Sex, and BMI) Spearman’s Rank Correlations Between the Biomarkers and St. George Respiratory Questionnaire (SGRQ)

| **Protein** | **Unadjusted**  **r-value** | **Unadjusted**  **p-value** | **Adjusted**  **r-value** | **Adjusted**  **p-value** |
| --- | --- | --- | --- | --- |
| Activin A | **0.26** | **0.001** | **0.23** | **0.003** |
| ADAMTS13 | -0.14 | 0.062 | **-0.19** | **0.016** |
| Eotaxin | 0.12 | 0.111 | **0.17** | **0.028** |
| Fas | 0.15 | 0.052 | 0.08 | 0.278 |
| GDF15 | **0.38** | **<0.001** | **0.40** | **<0.001** |
| GROα | 0.02 | 0.754 | 0.02 | 0.781 |
| ICAM1 | **0.24** | **0.002** | **0.25** | **0.001** |
| IL1α | 0.01 | 0.904 | 0.04 | 0.652 |
| IL6 | 0.14 | 0.069 | 0.11 | 0.158 |
| IL7 | **0.21** | **0.005** | **0.19** | **0.015** |
| IL8 | 0.13 | 0.088 | 0.13 | 0.097 |
| IL10 | 0.10 | 0.215 | 0.14 | 0.068 |
| IL15 | -0.05 | 0.535 | 0.02 | 0.817 |
| MCP1 | **0.34** | **<0.001** | **0.34** | **<0.001** |
| MDC | **0.25** | **0.001** | **0.23** | **0.003** |
| MIP1α | 0.02 | 0.801 | -0.01 | 0.910 |
| MIP1β | -0.06 | 0.461 | -0.02 | 0.751 |
| MMP1 | **0.16** | **0.034** | **0.18** | **0.020** |
| MMP7 | **0.41** | **<0.001** | **0.42** | **<0.001** |
| MMP9 | 0.06 | 0.449 | 0.02 | 0.800 |
| MPO | **0.19** | **0.014** | 0.13 | 0.084 |
| OPN | -0.05 | 0.500 | -0.06 | 0.438 |
| PAI1 | 0.11 | 0.158 | 0.06 | 0.432 |
| PARC | **0.38** | **<0.001** | **0.37** | **<0.001** |
| RAGE | **-0.26** | **0.001** | **-0.26** | **0.001** |
| RANTES | **0.16** | **0.034** | 0.14 | 0.071 |
| SOST | -0.09 | 0.244 | -0.11 | 0.167 |
| TARC | **0.32** | **<0.001** | **0.33** | **<0.001** |
| TNFR1 | 0.22 | 0.005 | 0.13 | 0.091 |
| TNFR2 | **0.23** | **0.003** | **0.17** | **0.030** |
| TNFα | -0.02 | 0.800 | -0.03 | 0.737 |
| VEGFA | 0.09 | 0.255 | 0.08 | 0.278 |

**Table S9**: Unadjusted and Adjusted (for Age, Sex, and BMI) Spearman’s Rank Correlations Between the Biomarkers and 12-Item Short Form Health Survey (SF-12)

| **Protein** | **Unadjusted**  **r-value** | **Unadjusted**  **p-value** | **Adjusted**  **r-value** | **Adjusted**  **p-value** |
| --- | --- | --- | --- | --- |
| Activin A | **-0.18** | **0.018** | -0.15 | 0.058 |
| ADAMTS13 | 0.11 | 0.141 | 0.14 | 0.063 |
| Eotaxin | -0.06 | 0.434 | -0.12 | 0.118 |
| Fas | -0.13 | 0.092 | -0.07 | 0.356 |
| GDF15 | **-0.23** | **0.002** | **-0.25** | **0.001** |
| GROα | -0.02 | 0.795 | -0.01 | 0.882 |
| ICAM1 | -0.14 | 0.081 | -0.15 | 0.058 |
| IL1α | -0.02 | 0.830 | -0.05 | 0.562 |
| IL6 | -0.10 | 0.218 | -0.07 | 0.368 |
| IL7 | -0.13 | 0.092 | -0.11 | 0.161 |
| IL8 | -0.10 | 0.208 | -0.09 | 0.228 |
| IL10 | -0.11 | 0.138 | **-0.17** | **0.029** |
| IL15 | 0.01 | 0.874 | -0.05 | 0.492 |
| MCP1 | **-0.24** | **0.002** | **-0.24** | **0.002** |
| MDC | **-0.16** | **0.035** | -0.14 | 0.075 |
| MIP1α | -0.05 | 0.548 | -0.03 | 0.693 |
| MIP1β | 0.10 | 0.186 | 0.06 | 0.420 |
| MMP1 | **-0.17** | **0.028** | **-0.19** | **0.016** |
| MMP7 | **-0.27** | **<0.001** | **-0.28** | **<0.001** |
| MMP9 | -0.02 | 0.769 | 0.01 | 0.925 |
| MPO | -0.12 | 0.123 | -0.07 | 0.383 |
| OPN | -0.03 | 0.686 | -0.03 | 0.747 |
| PAI1 | -0.12 | 0.125 | -0.08 | 0.313 |
| PARC | **-0.27** | **0.001** | **-0.25** | **0.001** |
| RAGE | **0.18** | **0.019** | **0.18** | **0.017** |
| RANTES | -0.13 | 0.100 | -0.11 | 0.164 |
| SOST | 0.14 | 0.065 | **0.15** | **0.049** |
| TARC | **-0.20** | **0.009** | **-0.22** | **0.004** |
| TNFR1 | **-0.16** | **0.035** | -0.08 | 0.320 |
| TNFR2 | **-0.23** | **0.003** | **-0.18** | **0.024** |
| TNFα | 0.02 | 0.777 | 0.03 | 0.745 |
| VEGFA | -0.06 | 0.406 | -0.06 | 0.430 |

**Table S10**: Unadjusted and Adjusted (for Age, Sex, and BMI) Spearman’s rank Correlations Between the Biomarkers and 6-Minute Walk Test (6MWT)

| **Protein** | **Unadjusted**  **r-value** | **Unadjusted**  **p-value** | **Adjusted**  **r-value** | **Adjusted**  **p-value** |
| --- | --- | --- | --- | --- |
| Activin A | -0.16 | 0.065 | -0.06 | 0.471 |
| ADAMTS13 | **0.24** | **0.004** | **0.20** | **0.020** |
| Eotaxin | 0.08 | 0.354 | 0.03 | 0.707 |
| Fas | -0.04 | 0.632 | 0.02 | 0.854 |
| GDF15 | **-0.21** | **0.013** | **-0.19** | **0.024** |
| GROα | -0.09 | 0.302 | -0.04 | 0.675 |
| ICAM1 | -0.14 | 0.099 | **-0.19** | **0.03** |
| IL1α | 0.00 | 0.967 | -0.03 | 0.713 |
| IL6 | **-0.30** | **<0.001** | **-0.29** | **0.001** |
| IL7 | 0.03 | 0.691 | 0.02 | 0.848 |
| IL8 | -0.05 | 0.544 | -0.05 | 0.569 |
| IL10 | -0.06 | 0.484 | -0.13 | 0.145 |
| IL15 | -0.03 | 0.739 | -0.06 | 0.488 |
| MCP1 | 0.00 | 0.990 | 0.00 | 0.988 |
| MDC | -0.04 | 0.613 | -0.06 | 0.510 |
| MIP1α | -0.07 | 0.409 | -0.08 | 0.336 |
| MIP1β | 0.06 | 0.455 | 0.03 | 0.764 |
| MMP1 | **-0.20** | **0.016** | **-0.20** | **0.018** |
| MMP7 | **-0.29** | **<0.001** | **-0.29** | **0.001** |
| MMP9 | -0.03 | 0.727 | -0.07 | 0.447 |
| MPO | -0.07 | 0.388 | -0.08 | 0.352 |
| OPN | -0.09 | 0.285 | -0.05 | 0.594 |
| PAI1 | -0.04 | 0.673 | -0.04 | 0.671 |
| PARC | -0.16 | 0.064 | -0.15 | 0.079 |
| RAGE | 0.14 | 0.098 | 0.16 | 0.061 |
| RANTES | -0.04 | 0.678 | -0.04 | 0.645 |
| SOST | 0.02 | 0.846 | 0.02 | 0.852 |
| TARC | -0.16 | 0.058 | **-0.20** | **0.023** |
| TNFR1 | -0.15 | 0.081 | -0.07 | 0.402 |
| TNFR2 | -0.11 | 0.194 | -0.05 | 0.543 |
| TNFα | -0.07 | 0.442 | -0.07 | 0.395 |
| VEGFA | -0.14 | 0.092 | -0.13 | 0.124 |

**Table S11**: LASSO Regression Analysis to Predict Forced Vital Capacity (FVC), Diffusing Capacity of the Lung for Carbon Monoxide (DLCO), and Forced Expiratory Volume in 1 Second (FEV1)

|  | **Coefficient** | | | **Importance** | | |
| --- | --- | --- | --- | --- | --- | --- |
| **Protein** | **FVC** | **DLCO** | **FEV1** | **FVC** | **DLCO** | **FEV1** |
| RAGE | 5.15 | 7.17 | 3.96 | 8.67 | 10.12 | 7.24 |
| MMP7 | -3.96 | -6.37 | -2.95 | 6.67 | 8.99 | 5.39 |
| MDC | -2.19 | -2.35 | -1.90 | 3.69 | 3.31 | 3.48 |
| PARC | -0.32 | -3.38 | -0.58 | 0.54 | 4.77 | 1.06 |
| Eotaxin | -1.34 | -0.73 | -1.07 | 2.26 | 1.02 | 1.95 |
| MCP1 | -1.69 | -0.86 | -0.60 | 2.84 | 1.21 | 1.09 |
| Activin A | -1.13 | -1.99 | 0.00 | 1.91 | 2.80 | 0.00 |
| MMP1 | 0.00 | -2.60 | 0.00 | 0.00 | 3.67 | 0.00 |
| TARC | -0.22 | -1.85 | 0.00 | 0.38 | 2.60 | 0.00 |
| VEGFA | 0.00 | 1.78 | 0.00 | 0.00 | 2.51 | 0.00 |
| IL8 | 0.00 | -1.59 | 0.00 | 0.00 | 2.24 | 0.00 |
| MMP9 | 0.00 | 1.51 | 0.00 | 0.00 | 2.13 | 0.00 |
| ICAM1 | 0.00 | -1.44 | 0.00 | 0.00 | 2.03 | 0.00 |
| IL10 | -0.30 | -0.05 | -0.29 | 0.50 | 0.07 | 0.53 |
| OPN | 0.00 | 0.71 | 0.00 | 0.00 | 1.00 | 0.00 |
| Fas | 0.00 | 0.50 | 0.00 | 0.00 | 0.71 | 0.00 |
| GDF15 | 0.00 | -0.46 | 0.00 | 0.00 | 0.64 | 0.00 |
| ADAMTS13 | 0.00 | 0.40 | 0.00 | 0.00 | 0.57 | 0.00 |
| MPO | -0.18 | 0.00 | 0.00 | 0.30 | 0.00 | 0.00 |
| TNFR1 | 0.00 | 0.02 | 0.00 | 0.00 | 0.03 | 0.00 |
| SOST | 0.00 | 0.02 | 0.00 | 0.00 | 0.02 | 0.00 |
| TNFα | 0.00 | 0.00 | 0.00 | 0.00 | 0.00 | 0.00 |
| PAI1 | 0.00 | 0.00 | 0.00 | 0.00 | 0.00 | 0.00 |
| IL6 | 0.00 | 0.00 | 0.00 | 0.00 | 0.00 | 0.00 |
| IL15 | 0.00 | 0.00 | 0.00 | 0.00 | 0.00 | 0.00 |
| GROα | 0.00 | 0.00 | 0.00 | 0.00 | 0.00 | 0.00 |
| TNFR2 | 0.00 | 0.00 | 0.00 | 0.00 | 0.00 | 0.00 |
| MIP1α | 0.00 | 0.00 | 0.00 | 0.00 | 0.00 | 0.00 |
| IL1α | 0.00 | 0.00 | 0.00 | 0.00 | 0.00 | 0.00 |
| MIP1β | 0.00 | 0.00 | 0.00 | 0.00 | 0.00 | 0.00 |
| IL7 | 0.00 | 0.00 | 0.00 | 0.00 | 0.00 | 0.00 |
| RANTES | 0.00 | 0.00 | 0.00 | 0.00 | 0.00 | 0.00 |

**Table S12**: LASSO Regression Analysis to Predict St. George Respiratory Questionnaire (SGRQ), 12-Item Short Form Health Survey (SF-12), and the 6-minute Walk Test (6MWT)

|  | **Coefficient** | | | **Importance** | | |
| --- | --- | --- | --- | --- | --- | --- |
| **Protein** | **SGRQ** | **SF-12** | **6MWT** | **SGRQ** | **SF-12** | **6MWT** |
| MMP7 | 3.77 | -0.91 | -12.41 | 5.34 | 2.44 | 3.86 |
| IL6 | 0.00 | -1.25 | -22.26 | 0.00 | 3.36 | 6.93 |
| PARC | 3.50 | -1.52 | 0.00 | 4.96 | 4.09 | 0.00 |
| MCP1 | 1.78 | -1.14 | 0.00 | 2.53 | 3.08 | 0.00 |
| TARC | 1.43 | -0.49 | 0.00 | 2.02 | 1.32 | 0.00 |
| MIP1β | 0.00 | 0.95 | 0.00 | 0.00 | 2.57 | 0.00 |
| SOST | 0.00 | 0.90 | 0.00 | 0.00 | 2.43 | 0.00 |
| TNFR2 | 0.00 | -0.78 | 0.00 | 0.00 | 2.09 | 0.00 |
| GROα | 0.00 | 0.72 | 0.00 | 0.00 | 1.95 | 0.00 |
| ADAMTS13 | 0.00 | 0.00 | 5.42 | 0.00 | 0.00 | 1.69 |
| TNFα | 0.00 | 0.34 | 0.00 | 0.00 | 0.91 | 0.00 |
| IL7 | 0.00 | -0.31 | 0.00 | 0.00 | 0.83 | 0.00 |
| PAI1 | 0.00 | -0.26 | 0.00 | 0.00 | 0.71 | 0.00 |
| MMP1 | 0.00 | -0.25 | 0.00 | 0.00 | 0.67 | 0.00 |
| Activin A | 0.00 | -0.17 | 0.00 | 0.00 | 0.45 | 0.00 |
| MPO | 0.00 | -0.01 | 0.00 | 0.00 | 0.03 | 0.00 |
| MDC | 0.00 | 0.00 | 0.00 | 0.00 | 0.00 | 0.00 |
| Eotaxin | 0.00 | 0.00 | 0.00 | 0.00 | 0.00 | 0.00 |
| IL15 | 0.00 | 0.00 | 0.00 | 0.00 | 0.00 | 0.00 |
| VEGFA | 0.00 | 0.00 | 0.00 | 0.00 | 0.00 | 0.00 |
| Fas | 0.00 | 0.00 | 0.00 | 0.00 | 0.00 | 0.00 |
| TNFR1 | 0.00 | 0.00 | 0.00 | 0.00 | 0.00 | 0.00 |
| MIP1α | 0.00 | 0.00 | 0.00 | 0.00 | 0.00 | 0.00 |
| IL1α | 0.00 | 0.00 | 0.00 | 0.00 | 0.00 | 0.00 |
| MMP9 | 0.00 | 0.00 | 0.00 | 0.00 | 0.00 | 0.00 |
| IL10 | 0.00 | 0.00 | 0.00 | 0.00 | 0.00 | 0.00 |
| RANTES | 0.00 | 0.00 | 0.00 | 0.00 | 0.00 | 0.00 |
| IL8 | 0.00 | 0.00 | 0.00 | 0.00 | 0.00 | 0.00 |
| GDF15 | 0.00 | 0.00 | 0.00 | 0.00 | 0.00 | 0.00 |
| OPN | 0.00 | 0.00 | 0.00 | 0.00 | 0.00 | 0.00 |
| RAGE | 0.00 | 0.00 | 0.00 | 0.00 | 0.00 | 0.00 |
| ICAM1 | 0.00 | 0.00 | 0.00 | 0.00 | 0.00 | 0.00 |

**Table S13**: Associations between Circulating Biomarkers and Risk of Death after adjustment for Age, BMI, and Sex in IPF Participants

| **Protein** | **HR** | **CI (lower HR)** | **CI (upper HR)** | **p-value** |
| --- | --- | --- | --- | --- |
| Activin A | **1.54** | **1.16** | **2.04** | **0.003** |
| IL8 | **1.49** | **1.14** | **1.95** | **0.004** |
| TARC | **1.34** | **1.06** | **1.70** | **0.016** |
| MDC | **1.37** | **1.04** | **1.80** | **0.023** |
| MMP7 | **1.40** | **1.04** | **1.88** | **0.025** |
| GDF15 | **1.42** | **1.04** | **1.93** | **0.026** |
| SOST | 0.71 | 0.50 | 1.02 | 0.062 |
| TNFR1 | 1.35 | 0.96 | 1.91 | 0.085 |
| GROα | 0.72 | 0.47 | 1.11 | 0.140 |
| TNFR2 | 1.26 | 0.92 | 1.73 | 0.142 |
| MMP1 | 1.26 | 0.92 | 1.72 | 0.145 |
| MCP1 | 1.18 | 0.91 | 1.53 | 0.211 |
| MPO | 1.20 | 0.90 | 1.59 | 0.220 |
| OPN | 0.79 | 0.51 | 1.20 | 0.269 |
| ICAM1 | 1.17 | 0.88 | 1.55 | 0.275 |
| ADAMTS13 | 0.83 | 0.59 | 1.17 | 0.283 |
| MMP9 | 1.18 | 0.85 | 1.63 | 0.330 |
| PARC | 0.88 | 0.67 | 1.15 | 0.345 |
| Eotaxin | 1.14 | 0.86 | 1.51 | 0.366 |
| IL7 | 0.88 | 0.67 | 1.16 | 0.371 |
| IL10 | 0.91 | 0.71 | 1.17 | 0.484 |
| TNFα | 0.89 | 0.64 | 1.24 | 0.491 |
| VEGFA | 0.90 | 0.65 | 1.26 | 0.549 |
| PAI1 | 1.08 | 0.83 | 1.41 | 0.569 |
| IL15 | 0.92 | 0.68 | 1.25 | 0.613 |
| RAGE | 0.93 | 0.63 | 1.37 | 0.720 |
| RANTES | 1.03 | 0.81 | 1.31 | 0.790 |
| MIP1β | 1.03 | 0.75 | 1.42 | 0.849 |
| IL1α | 0.98 | 0.76 | 1.26 | 0.876 |
| Fas | 1.02 | 0.77 | 1.35 | 0.892 |
| IL6 | 1.01 | 0.73 | 1.38 | 0.967 |
| MIP1α | 1.01 | 0.75 | 1.35 | 0.973 |

Abbreviations: HR = Hazard Ratio; CI = Confidence Interval.

**Table S14**: Unadjusted and Adjusted (for Age, Sex, and BMI) Spearman’s Rank Correlations between the Circulating Biomarkers and *P16* Lung Gene Expression

| **Protein** | **Unadjusted**  **r-value** | **Unadjusted**  **p-value** | **Unadjusted**  **r-value** | **Unadjusted**  **p-value** |
| --- | --- | --- | --- | --- |
| Activin A | **0.34** | **<0.001** | **0.30** | **<0.001** |
| ADAMTS13 | **-0.17** | **0.035** | -0.11 | 0.182 |
| Eotaxin | 0.13 | 0.106 | 0.12 | 0.144 |
| Fas | -0.09 | 0.283 | -0.14 | 0.104 |
| GDF15 | **0.32** | **<0.001** | **0.29** | **<0.001** |
| GROα | 0.06 | 0.478 | 0.00 | 0.987 |
| ICAM1 | **0.25** | **0.003** | **0.24** | **0.004** |
| IL1α | -0.13 | 0.115 | -0.14 | 0.101 |
| IL6 | 0.04 | 0.671 | 0.05 | 0.567 |
| IL7 | 0.07 | 0.430 | 0.10 | 0.211 |
| IL8 | **0.19** | **0.024** | **0.17** | **0.040** |
| IL10 | -0.01 | 0.932 | -0.03 | 0.695 |
| IL15 | -0.05 | 0.551 | -0.09 | 0.284 |
| MCP1 | **0.23** | **0.005** | **0.22** | **0.007** |
| MDC | **0.23** | **0.006** | **0.25** | **0.003** |
| MIP1α | 0.02 | 0.789 | 0.01 | 0.929 |
| MIP1β | -0.10 | 0.250 | -0.11 | 0.209 |
| MMP1 | **0.24** | **0.003** | **0.23** | **0.006** |
| MMP7 | **0.30** | **<0.001** | **0.29** | **<0.001** |
| MMP9 | -0.06 | 0.453 | -0.01 | 0.906 |
| MPO | 0.05 | 0.574 | 0.11 | 0.196 |
| OPN | 0.01 | 0.900 | -0.04 | 0.629 |
| PAI1 | 0.06 | 0.468 | 0.10 | 0.223 |
| PARC | **0.17** | **0.035** | **0.18** | **0.029** |
| RAGE | 0.03 | 0.748 | 0.00 | 0.979 |
| RANTES | 0.03 | 0.746 | 0.05 | 0.528 |
| SOST | -0.07 | 0.381 | -0.07 | 0.436 |
| TARC | **0.18** | **0.031** | 0.16 | 0.061 |
| TNFR1 | 0.05 | 0.510 | 0.04 | 0.606 |
| TNFR2 | 0.12 | 0.137 | 0.10 | 0.224 |
| TNFα | -0.03 | 0.687 | -0.06 | 0.486 |
| VEGFA | 0.11 | 0.198 | 0.06 | 0.476 |

**Table S15**: LASSO Regression Analysis to Predict Lung *P16* Gene Expression

| **Protein** | **Coefficient** | **Importance** |
| --- | --- | --- |
| Activin A | 0.199 | 6.788 |
| IL1α | -0.104 | 3.535 |
| Fas | -0.102 | 3.461 |
| MMP7 | 0.083 | 2.813 |
| Eotaxin | 0.068 | 2.328 |
| MCP1 | 0.065 | 2.202 |
| MDC | 0.063 | 2.146 |
| ADAMTS13 | -0.044 | 1.490 |
| RAGE | 0.018 | 0.597 |
| TNFR2 | 0.010 | 0.356 |
| IL8 | 0.008 | 0.267 |
| PARC | 0.006 | 0.202 |
| MMP1 | 0.002 | 0.063 |
| TARC | 0.000 | 0.000 |
| GDF15 | 0.000 | 0.000 |
| RANTES | 0.000 | 0.000 |
| IL10 | 0.000 | 0.000 |
| OPN | 0.000 | 0.000 |
| IL7 | 0.000 | 0.000 |
| TNFR1 | 0.000 | 0.000 |
| GROα | 0.000 | 0.000 |
| PAI1 | 0.000 | 0.000 |
| SOST | 0.000 | 0.000 |
| VEGFA | 0.000 | 0.000 |
| MIP1β | 0.000 | 0.000 |
| MPO | 0.000 | 0.000 |
| TNFα | 0.000 | 0.000 |
| ICAM1 | 0.000 | 0.000 |
| MIP1α | 0.000 | 0.000 |
| MMP9 | 0.000 | 0.000 |
| IL6 | 0.000 | 0.000 |
| IL15 | 0.000 | 0.000 |
